# Novel inhibition of *Staphylococcus aureus* sortase A by plantamajoside: implications for controlling multidrug-resistant infections

**DOI:** 10.1128/aem.01804-24

**Published:** 2024-12-31

**Authors:** Yujia Chen, Wei Li, Li Wang, Bingmei Wang, Jian Suo

**Affiliations:** 1 Department of Gastrocolorectal Surgery, General Surgery Center, The First Hospital of Jilin University 117971 , Changchun, Jilin, China; 2 Clinical Medical College, Changchun University of Chinese Medicine 159345 , Changchun, Jilin, China; Kyoto University, Kyoto, Japan

**Keywords:** methicillin-resistant *Staphylococcus aureus*, sortase A, antivirulence, plantamajoside

## Abstract

In confronting the significant challenge posed by multidrug-resistant (MDR) pathogens, particularly methicillin-resistant *Staphylococcus aureus* (MRSA), the development of innovative anti-infective strategies is essential. Our research focuses on sortase A (SrtA), a vital enzyme for anchoring surface proteins in *S. aureus*. We discovered that plantamajoside (PMS), a phenylpropanoid glycoside extracted from *Plantago asiatica L*. (Plantaginaceae), acts as an effective and reversible inhibitor of SrtA, with a notable IC_50_ value of 22.93 µg/mL. This breakthrough provides a novel approach to combat both resistance and virulence in MRSA. PMS significantly inhibits *S. aureus* adhesion to fibrinogen, reducing biofilm formation and hindering the anchoring of staphylococcal protein A to the cell wall. Live-dead cell assays demonstrated increased survival rates in PMS-treated MRSA-infected A549 cells. Fluorescence quenching experiments revealed a robust interaction between PMS and SrtA, with mechanistic analyses pinpointing the critical R197 amino acid residue as the target site. *In vivo*, PMS was highly effective in a *Galleria mellonella* infection model, reducing mortality rates in MRSA-infected larvae. Additionally, PMS demonstrated therapeutic efficacy in a mouse pneumonia model, improved survival rates, reduced the bacterial load in pulmonary tissues, and mitigated lung damage. These results validate PMS as a promising compound to mitigate MRSA virulence and thwart resistance by targeting SrtA. This study highlights PMS as a leading candidate for controlling MRSA infections, showing the potential of targeting specific bacterial mechanisms in the fight against MDR infections.

**IMPORTANCE:**

The increasing issue of antibiotic resistance, particularly in methicillin-resistant *Staphylococcus aureus* (MRSA), demands innovative solutions. Our study presents plantamajoside (PMS) as a novel inhibitor of sortase A (SrtA), a key enzyme in *S. aureus* pathogenicity. By targeting SrtA, PMS shows promise in curbing the ability of MRSA to adhere, invade, and form biofilms, thereby reducing its virulence without exerting selective pressure for resistance. This research is significant because it introduces a potential new strategy in the antimicrobial arsenal, aligning with the global effort to combat drug-resistant infections. This study is crucial because it identifies a natural compound that can reduce the harmful effects of MRSA, a type of bacteria that is very hard to treat owing to resistance to many antibiotics. This discovery could lead to new treatments that are less likely to cause bacteria to become resistant, which is a major win in the fight against infections that are difficult to cure.

## INTRODUCTION

*Staphylococcus aureus* (*S. aureus*), commonly referred to as golden staph, is a significant opportunistic pathogen in the realm of global health owing to its contribution to a spectrum of human infections (1). These conditions range from mild skin irritation to severe, life-threatening conditions such as necrotizing pneumonia (2, 3). According to the findings of the 2022 National Bacterial Drug Resistance Surveillance Report, among the 2,000 hospitals monitored, *S. aureus* emerged as the predominant pathogen, accounting for 32.3% of clinical isolates among gram-positive bacteria (4). The global health implications of this bacterium are profound, considering its pivotal role in the escalating crisis of antibiotic resistance, which poses a substantial threat to public health worldwide.

Furthermore, the zoonotic potential of *S. aureus*, including methicillin-resistant *S. aureus* (MRSA), highlights its importance beyond human infections. This pathogen not only affects human populations but also plays a critical role in veterinary medicine. The horizontal exchange of antimicrobial resistance genes between humans, animals, and even the environment exacerbates the challenge of controlling their spread. In light of this, the One Health approach, which integrates human, animal, and environmental health, has become increasingly essential. This perspective emphasizes the need for holistic strategies to mitigate antimicrobial resistance across species and ecosystems. As such, our study seeks to explore innovative interventions to curb the spread of MRSA, contributing to a broader understanding of resistance transmission and control strategies.

The conventional approach for treating bacterial infections predominantly relies on antibiotics, which target bacterial growth by inhibiting essential protein synthesis or disrupting key components such as the cell wall (5). However, this strategy has led to significant evolutionary pressure on bacteria, resulting in the emergence of antibiotic-resistant strains. This growing trend of antibiotic resistance is a mounting global challenge, underscoring the urgent need for novel therapeutic strategies. Moreover, the widespread use of broad-spectrum antibiotics has unintended consequences for ecosystems and host microbiomes. These antibiotics can disrupt the delicate balance of microbial communities, leading to long-term ecological impacts and health issues such as dysbiosis, diarrhea, and intestinal infections in the host. This scenario further necessitates the exploration of alternative antimicrobial strategies (6).

*S. aureus* primarily employs adhesins and toxins as virulence factors to establish infections or induce pathological changes in a variety of hosts, including humans and animals. Adhesins, covalently anchored proteins on the cell surface, play crucial roles in mediating infection processes, such as attachment, colonization, cell‒cell interactions, and invasion of host tissues (7). Disrupting the anchoring of these virulence-associated proteins has emerged as a promising approach for combating *S. aureus* infections, given that these factors are nonessential for bacterial survival (8). LPXTG motif-containing cell surface proteins are secreted and covalently anchored to the peptidoglycan layer of the gram-positive cell wall, a process mediated by sortase enzymes (9, 10). Sortase A (SrtA), a cysteine transpeptidase, is central to this process and has emerged as an ideal target for novel anti-MRSA (methicillin-resistant *Staphylococcus aureus*) drug development. Biofilm formation, which causes nonspecific antibiotic resistance, plays a crucial role in many biofilm-related *S. aureus* infections. Various surface proteins anchored by SrtA contribute to the formation of biofilms. These surface proteins also influence *S. aureus* adhesion to the host, invasion, and immune evasion (11). Inhibiting SrtA minimally affects bacterial growth, thereby reducing the selective pressure for resistance development (12, 13). This approach aligns with the emerging paradigm of antimicrobial strategies, which focus on attenuating virulence rather than bacterial eradication, potentially offering a viable solution to the pressing issue of antibiotic resistance.

The millennium-old medicinal value of plants has gained renewed interest in the context of antibiotic-resistant pathogens. Plant extracts, which are rich in biodegradable secondary metabolites such as phenols, flavonoids, saponins, and alkaloids, are promising alternatives because of their antibacterial, anti-inflammatory, wound-healing, and antiviral properties (14). These properties potentially reduce the likelihood of disease occurrence, representing a sustainable approach for addressing the antibiotic resistance crisis (15).

**Fig 1:**
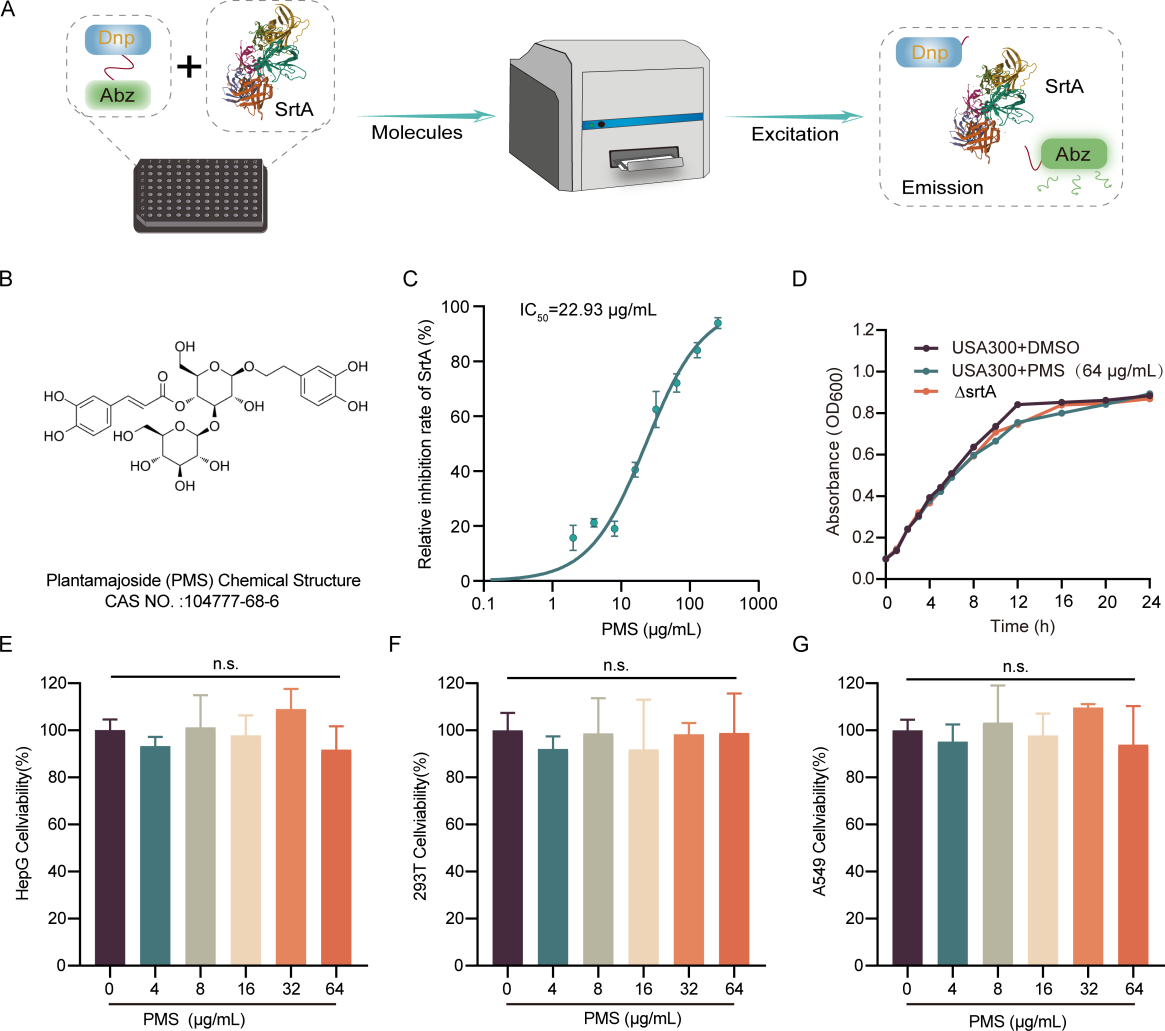
Inhibitory effect of PMS on the SrtA enzyme. (**A**) Illustration of the inhibitor screening approach, wherein SrtA-mediated cleavage of the Abz-LPATG-Dap (Dnp)-NH_2_ substrate induces an energy shift, causing a fluorescence change. (**B**) Molecular structure of PMS. (**C**) PMS inhibited SrtA activity in a dose-dependent manner on the substrate Abz-LPATG-Dap (Dnp)-NH_2_, with an IC_50_ value of 22.93 µg/mL. (**D**) Growth curves for *S. aureus* USA 300 and a strain with the *srtA* mutation, both treated and not treated with PMS (64 µg/mL). (**E**) Cell viability percentages of the A549, HepG2, and 293T cell lines after 24 h of treatment with PMS at concentrations ranging from 0 to 64 µg/mL, as assessed by the CCK-8 method.

Our research focuses on PMS (illustrated in Fig. 1B), a phenylpropanoid glycoside isolated from *Plantago asiatica L*., known for its extensive use in traditional medicine. Plantamajoside (PMS) has been recognized for its antibacterial, antidiabetic, spasmolytic, antiviral, anti-inflammatory, and wound-healing properties (16, 17). Importantly, prior studies have shown that PMS plays a protective role in mouse models of lipopolysaccharide-induced acute lung injury, underscoring its potential utility in the study of pulmonary inflammation (18). This backdrop of multifunctional therapeutic effects sets the stage for our investigation into the unexplored impact of PMS on SrtA. We aimed to validate the anti-SrtA activity of PMS, elucidate its mechanism, and assess its antimicrobial efficacy *in vivo*, proposing it as a novel candidate for the fight against MRSA. This exploration not only addresses an immediate public health concern but also contributes significantly to the global effort in developing sustainable and effective antimicrobial therapies.

## RESULTS

### Confirmation of PMS as an inhibitor of *S. aureus* SrtA

To ensure the accuracy of our inhibitor screening, we used high-purity SrtA, as confirmed in Fig. S1B. The activity of SrtA was monitored via a fluorescence resonance energy transfer (FRET) assay. This assay relies on the principle that when SrtA actively cleaves its specific substrate, the associated donor and acceptor fluorophores are separated, leading to a detectable shift in the fluorescence signal, as illustrated in Fig. 1A. Notably, our results revealed that PMS significantly reduces the enzymatic activity of SrtA in a concentration-dependent manner. This inhibitory effect was quantified, with PMS achieving an IC_50_ value of 22.93 µg/mL (35.76 µM), as shown in Fig. 1C.

To address the issue of antimicrobial resistance, the antimicrobial properties of PMS were further evaluated. The minimum inhibitory concentration (MIC) against *S. aureus* USA300 was 512 µg/mL, indicating minimal antibacterial activity. Growth curve analysis revealed that cultures of *S. aureus* USA300 treated with 64 µg/mL PMS presented a growth pattern akin to that of untreated cultures (Fig. 1D), which is consistent with strategies focused on targeting virulence factors as a means to combat resistance while minimally impacting bacterial viability.

Additionally, the cytotoxicity of PMS at a concentration of 64 µg/mL was assessed in mammalian cell lines, including A549, HepG2, and 293T cells (Fig. 1E through G). The safety profile of PMS was rigorously evaluated via the *Galleria mellonella* larval infection model, a respected and increasingly adopted organism for pharmacological toxicity and efficacy studies. In this model, larvae treated with PMS at concentrations of 25 and 50 mg/kg presented no adverse effects, such as melanization or mortality (Fig. S3). The absence of detrimental effects at these significant concentrations highlights the biocompatibility and therapeutic potential of PMS.

In addition, the screening of PMS for PAINS via the SwissADME database revealed no PAINS-related structural alerts. These findings indicate that PMS does not possess any known structural motifs that could lead to false positive results in biochemical assays, supporting its validity as a potential SrtA inhibitor. The absence of PAINS-related issues further strengthens the reliability of our findings in subsequent analyses.

### Inhibitory effects of PMS on SrtA and virulence factors in *S. aureus*

**Fig 2:**
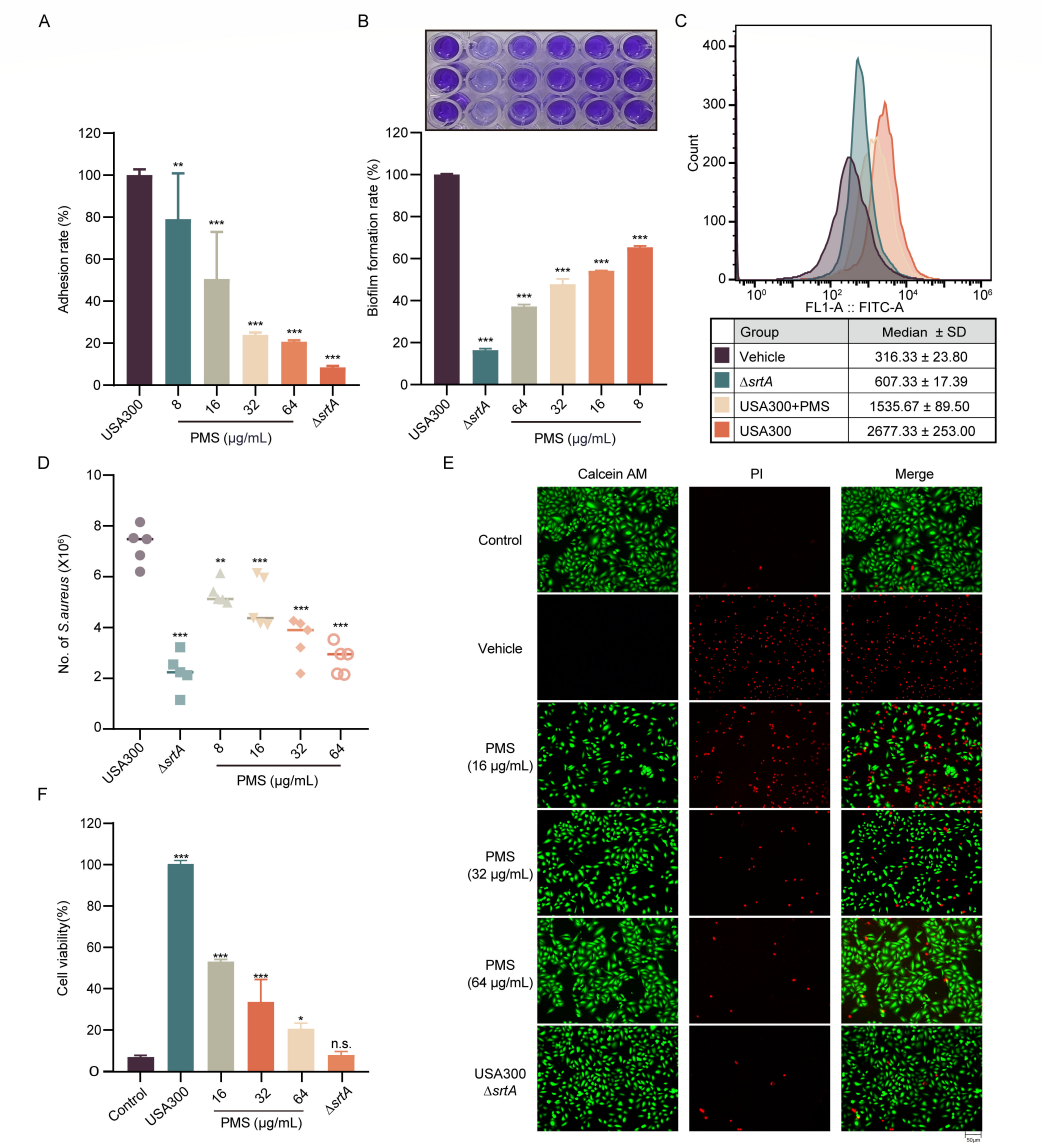
PMS modulates the virulence traits linked to SrtA in *S. aureus.* (**A**) The effect of PMS on the ability of *S. aureus* USA300 to adhere to fibrinogen was investigated. (**B**) Assessment of biofilm formation by *S. aureus* when exposed to PMS via crystal violet staining for quantitative analysis. (**C**) Surface protein (SpA) expression on *S. aureus* was investigated via the use of fluorescein isothiocyanate (FITC)-conjugated rabbit IgG for flow cytometry, with the Δ*srtA* variant serving as a benchmark. (**D**) PMS-mediated inhibition of *S. aureus* USA300 internalization into A549 cells. (**E and F**) Live‒dead cell staining and lactate dehydrogenase (LDH) release assays revealed that PMS mitigated the cytotoxic impact of MRSA on A549 cells in a dose-dependent manner. For Fig. 2B, D and F, statistical significance was determined by comparing each group to the wild-type (USA300) control group via one-way analysis of variance (ANOVA). **P < 0.01; ***P < 0.001; n.s. means not statistically significant.

Given the pivotal role of SrtA in bacterial adhesion and invasion, our initial investigations focused on evaluating the influence of PMS on bacterial adherence to fibrinogen. The data demonstrated that a notable dose-dependent decrease in fibrinogen adhesion correlated with increased PMS concentrations, culminating in a substantial reduction to 20.66% at 64 µg/mL (Fig. 2A).

Considering the critical functions of biofilms, complex microbial communities adhering to surfaces, antimicrobial resistance, and the established link between SrtA deficiency and impaired biofilm formation, we assessed the effect of PMS on biofilm development via crystal violet staining. In contrast to the robust biofilm formation observed in the control group, increasing the PMS concentration consistently attenuated this effect, with a pronounced decrease to 21.49% at a PMS concentration of 64 µg/mL.

Additionally, SrtA is implicated in modulating various cell wall-anchored surface proteins, particularly staphylococcal protein A (SpA), which interacts with the Fcγ and Fab domains of host immunoglobulins. By employing fluorescein isothiocyanate (FITC)-IgG antibody fluorescence as an indicator, we documented a dose-responsive decrease in the presence of SpA on the bacterial cell wall subsequent to PMS treatment, which is indicative of reduced SpA anchoring (Fig. 2C). This study was further extended to examine the impact of PMS on bacterial invasion via A549 cell invasion assays. The results indicated a marked reduction in the invasion of *S. aureus* into A549 cells after PMS treatment (Fig. 2D). Additionally, live/dead cell assays and lactate dehydrogenase (LDH) experiments further confirmed the dose-dependent protective effect of PMS on MRSA-infected A549 cells (Fig. 2E).

In conclusion, PMS significantly inhibits SrtA and its related pathogenic characteristics, including adhesion to fibrinogen, invasion of A549 cells, biofilm formation, and SpA anchoring to the cell wall. These insights underscore the potential of PMS as a therapeutic intervention against *S. aureus* infections by effectively targeting critical virulence determinants.

### PMS and SrtA exhibit direct binding interactions

**Fig 3:**
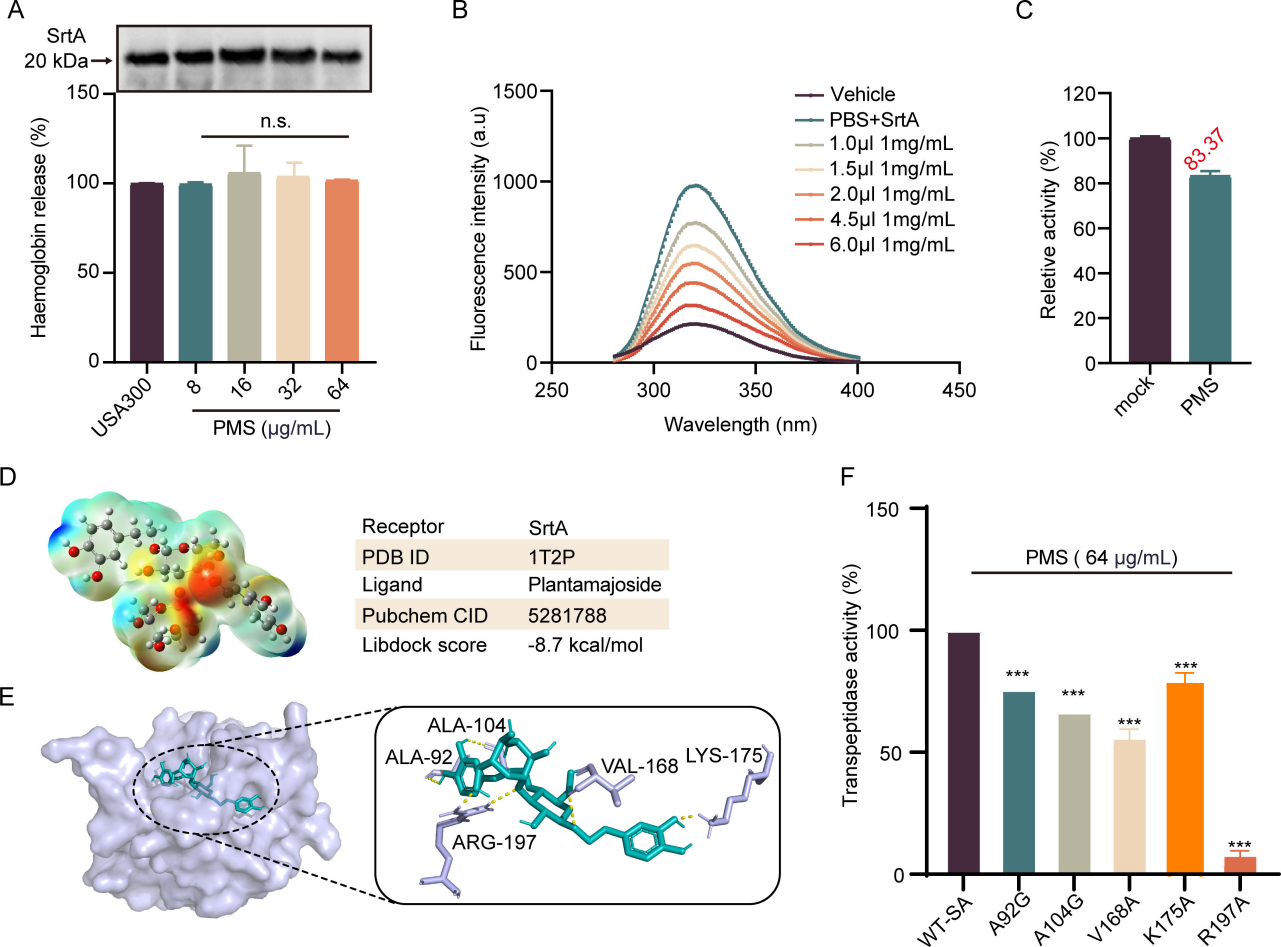
Investigating the direct interaction of PMSs with SrtA. (**A**) Protein blotting of SrtA from *S. aureus* exposed to PMS at concentrations ranging from 0 to 64 µg/mL. n.s. indicates not statistically significant according to one-way ANOVA. (**B**) Assessment of the interaction between PMS and SrtA via the fluorescence quenching method, demonstrating a stepwise reduction in SrtA fluorescence with increasing PMS concentration. (**C**) Treatment of SrtA with 10 × IC_50_ PMS, followed by dilution and FRET analysis for activity measurement, with the untreated SrtA (control) normalized to 100% activity. (**D**) Three-dimensional electrostatic potential mapping of PMS depicted through ABEE visualization. (**E**) The interaction dynamics between PMS and SrtA elucidated through molecular docking, indicating a total binding free energy of −8.7 kcal/mol. (**F**) The impact of PMS on the enzymatic activity of SrtA and its variants was analyzed through FRET. ***P < 0.001, calculated via one-way ANOVA. Three independent experiments were used for analysis.

To investigate the interaction between PMS and SrtA in *S. aureus*, various concentrations of PMS (0–64 µg/mL) were administered. Western blot analysis revealed consistent SrtA expression levels across all PMS concentrations, indicating that PMS does not markedly affect SrtA expression (Fig. 3A). Additionally, fluorescence quenching assays were utilized to probe the direct interaction between PMS and SrtA. The decrease in fluorescence intensity upon PMS binding suggested a specific interaction, with a binding constant (*K*_A_) of 8.17 × 10^4^ L/mol determined from fluorescence attenuation, confirming direct binding (Fig. 3B).

To characterize the nature of the PMS-SrtA interaction, a reversibility assay was conducted. The assay is designed such that a recovery of SrtA activity above 60% posttreatment typically indicates reversible, noncovalent binding. Treatment with PMS resulted in 83.37% recovery of SrtA activity relative to that of the control, indicating that PMS functions as a reversible inhibitor (see Fig. 3C). For an in-depth analysis of the PMS-SrtA interaction, the initial steps involved ABEE molecular electrostatic potential optimization to identify the optimal conformation of PMS. In Fig. 3D, the various colored spheres represent distinct regions of electrostatic potential distribution on the PMS molecule. The red regions indicate areas of negative charge density, which are predicted to form strong electrostatic interactions with the positively charged residues of SrtA, thereby increasing the binding affinity between PMS and SrtA. Conversely, the blue regions correspond to areas of positive charge density, which may interact with negatively charged regions of SrtA, further stabilizing the PMS-SrtA complex. These optimized electrostatic interactions facilitate PMS binding to the active site of SrtA, ultimately inhibiting its enzymatic activity. Molecular docking studies revealed multiple hydrogen bonds between PMS and SrtA, with the total free energy of binding calculated to be −8.7 kcal/mol, indicating strong affinity (Fig. 3E). To further elucidate this interaction, point mutations were introduced at key amino acid residues of SrtA (A92G, A104G, V168A, K175A, and R197A). Subsequent assays with 64 µg/mL PMS in these mutants revealed a significant reduction in SrtA activity. In contrast, wild-type SrtA treated with PMS presented distinctly different activity profiles (Fig. 3F). These results validate our computational predictions, identifying these amino acid sites as crucial for the binding efficacy of PMS to SrtA.

### Efficacy of PMS in treating MRSA infections: Insights from the *Galleria mellonella* (*G. mellonella*) model

**Fig 4:**
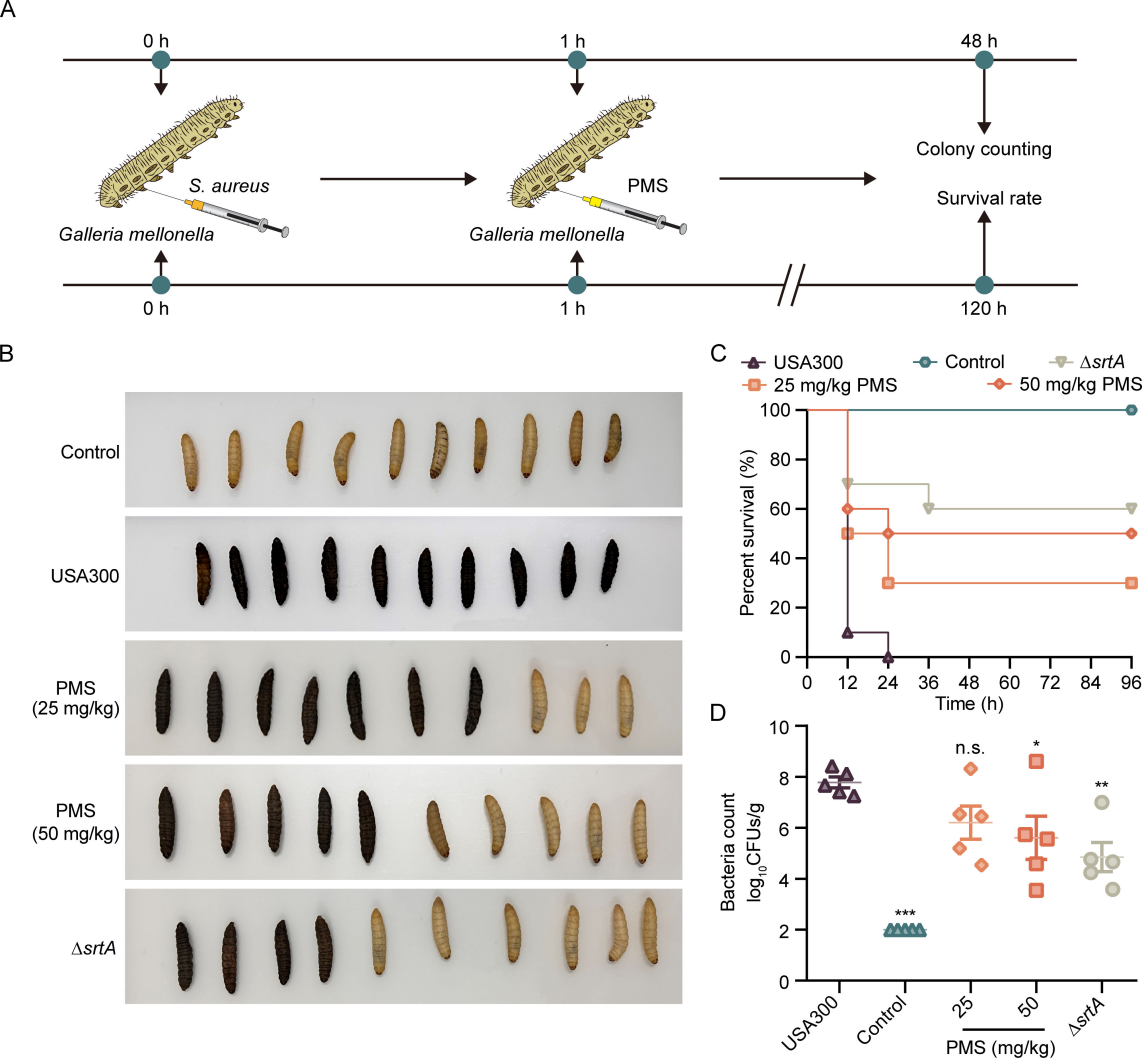
Efficacy of PMS in enhancing *G. mellonella* survival against MRSA. (**A**) Illustrative timeline of the MRSA infection process and assessment approaches in the *G. mellonella* model. In panel (**B**), a comparative study revealed different *G. mellonella* groups (each with 10 larvae), comprising an untreated control group, an *S. aureus* USA300-infected group, and groups receiving low (25 mg/kg) or high (50 mg/kg) doses of PMS. The survival rates of these groups were recorded at 120 h after infection. (**C**) This panel quantifies the colony-forming units (CFUs) in *G. mellonella* larvae postinfection (*n* = 5) via the agar dilution method. (**D**) The bacterial load of *G. mellonella* larvae. *P < 0.05; **P < 0.01; ***P < 0.001; n.s., not statistically significant; calculated via one-way ANOVA.

Owing to its biological similarities to other organisms and its ease of use in experiments, *G. mellonella* has emerged as an increasingly valuable model for testing new therapeutic agents. In this study, we investigated the efficacy of PMS, a promising compound, against MRSA infections. We infected *G. mellonella* larvae with the MRSA USA300 strain and subsequently administered PMS at various concentrations (25 and 50 mg/kg). Over a 5-day period, we closely monitored larval survival, color changes, and the bacterial load to evaluate the therapeutic effectiveness of PMS (Fig. 4A).

Our results revealed that the untreated control group experienced a significant decline in survival, with a 0% survival rate. In stark contrast, the groups treated with PMS presented considerable improvements in survival rates: 30% at 25 mg/kg and 50% at 50 mg/kg. The untreated larvae exhibited significant melanization, a sign of severe infection. However, larvae treated with PMS presented markedly reduced signs of infection (Fig. 4B and C).

With respect to the bacterial load, a notable decrease in the MRSA count was observed in the PMS-treated larvae, with the most significant reduction occurring at the 50 mg/kg dose (Fig. 4D). The *G. mellonella* model has effectively demonstrated the therapeutic potential of PMS against MRSA.

### PMS protects mice from MRSA-induced pneumonia

**Fig 5:**
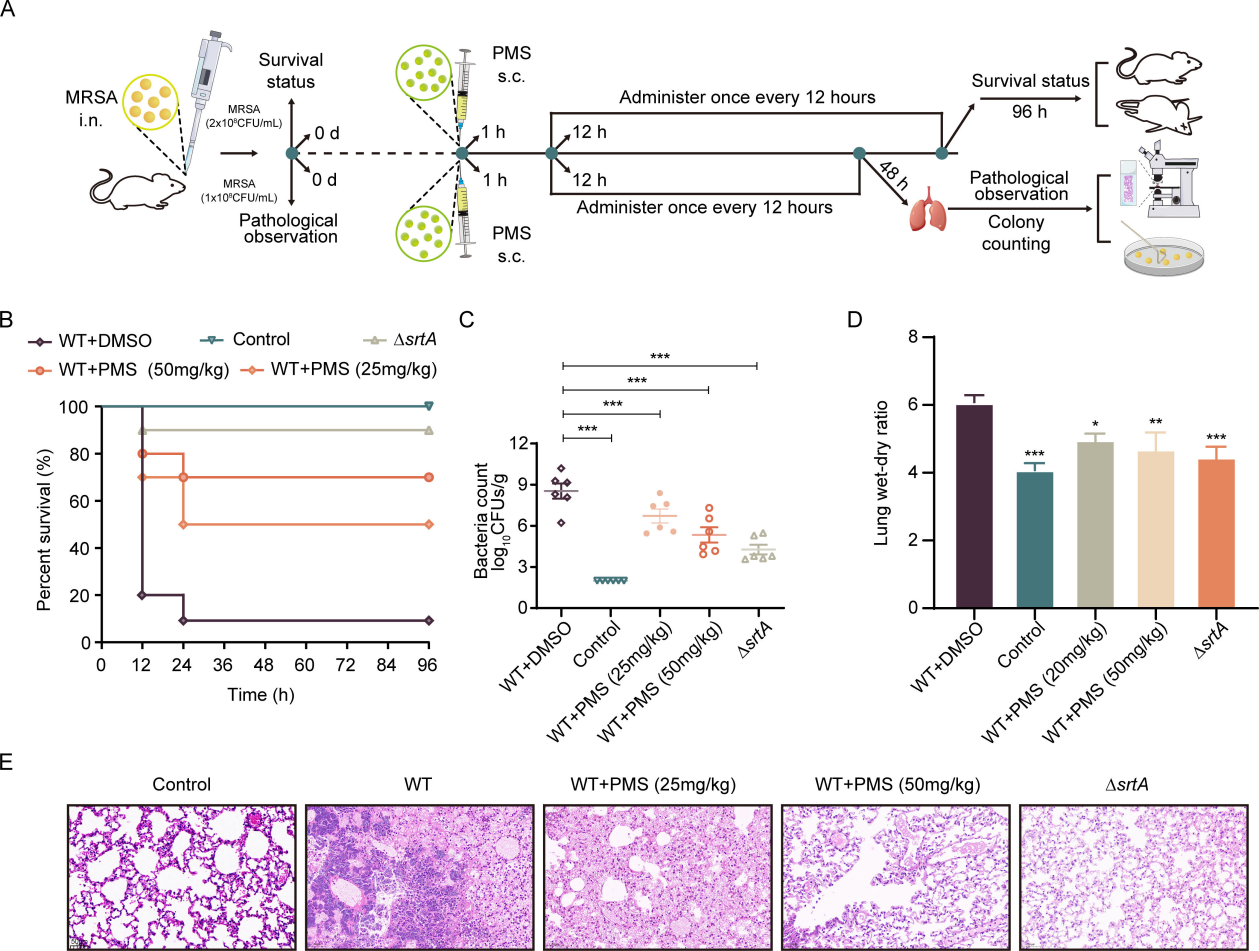
Effectiveness of PMS in the treatment of *S. aureus*-induced pneumonia in mice. (**A**) Illustration of the establishment of a pneumonia model in mice through nasal administration of MRSA, followed by an assessment of survival rates and pathological alterations. (**B**) The influence of PMS on the survival rate of mice (*n* = 10) subjected to a lethal dose of *S. aureus*, where a marked increase in survival was noted in the PMS-treated group compared with the wild-type (WT) group. (**C**) A decrease in the bacterial load in the lungs of mice (*n* = 6) treated with PMS at doses of 25 or 50 mg/kg was detected, with a significant reduction in bacterial levels in the PMS group relative to those in the WT group (*P < 0.05; **P < 0.01; ***P < 0.001 compared with the DMSO group). (**D**) Evaluation of the lung dry-to-wet weight ratio as an indicator of the therapeutic efficacy of PMS in MRSA-induced pneumonia in mice. **P < 0.01; n.s., not statistically significant, according to one-way ANOVA. (**E**) Details of both macroscopic and microscopic lung changes in mice, contrasting those treated with PMS (25 or 50 mg/kg) with those in the control group, accompanied by histopathological lung examinations. The scale bar represents 50 µm.

To evaluate the protective effects of PMS against pneumonia, a murine model of *S. aureus-induced* lung infection was established. This involves the intranasal administration of lethal or sublethal doses of MRSA to induce pneumonia. Subsequently, PMS was administered subcutaneously at 1 h postinfection and then every 12 h to assess its efficacy in mitigating pneumonia severity (Fig. 5A). The results indicated successful establishment of the MRSA-induced pneumonia model, with the untreated group exhibiting a survival rate of merely 10%. However, the administration of PMS at a dose of 50 mg/kg significantly increased the survival rate to 70%, especially when PMS was administered early in the infection course (Fig. 5B). This therapeutic effect of PMS was particularly evident in the acute pulmonary infection model.

Additionally, the impact of PMS on lung damage was assessed by intranasally administering a sublethal concentration of *S. aureus* to mice. Posttreatment, a marked decrease in the bacterial load in the lungs of PMS-treated mice was observed, decreasing from 8.74 ± 1.02 log10 CFU/g to 5.27 ± 1.32 log10 CFU/g (Fig. 5C). Histopathological examination of lung tissues from the infected group revealed significant inflammatory cell infiltration, which was noticeably mitigated following PMS treatment (Fig. 5E). Moreover, analysis of the lung wet‒dry weight ratios suggested that PMS effectively alleviated lung injury in mice with MRSA pneumonia (Fig. 5D). In summary, this study underscores the therapeutic potential of PMS for treating *S. aureus*-induced pneumonia in a murine model. These results emphasize the role of PMS in enhancing survival, reducing pulmonary damage, and lowering the bacterial load, indicating its promising application in the management of bacterial pneumonia.

### Synergistic enhancement of the efficacy of vancomycin against MRSA by PMS

The checkerboard assay results revealed a synergistic effect between PMS and vancomycin against MRSA USA300, with a fractional inhibitory concentration index of 0.375, indicating a pronounced increase in antibacterial activity when both agents were combined. Specifically, the MIC of vancomycin against USA300, which is typically 4 µg/mL, was significantly reduced to 1 µg/mL in the presence of 64 µg/mL (1/8 MIC) PMS. These findings demonstrate that PMS markedly increases the susceptibility of MRSA to vancomycin, highlighting its role as an effective potentiator (Table S3). The reduction in the MIC suggests that lower doses of vancomycin could be used in treatment, which may help mitigate the risk of adverse effects associated with higher antibiotic dosages.

## DISCUSSION

The emergence and exacerbation of antibiotic resistance are predominantly attributed to the overuse and misuse of antibiotics. In medical settings, prolonged or improper use of antibiotics prompts bacteria to develop resistance through natural selection. Moreover, the extensive use of antibiotics in agriculture has compounded this issue, leading to the spread of antibiotic resistance genes in the environment. The increase in resistance has resulted in a reduction in treatment options, increased treatment costs, and a decrease in patient recovery rates. Without intervention, antibiotic resistance is estimated to cause more than 10 million deaths annually by 2050, with medical costs increasing to $100 trillion (19). To address this escalating crisis, novel therapies that can replace current broad-spectrum antibiotics, such as those that selectively target pathogenic bacteria without altering the composition of neighboring microbial communities, are imperative (20). These new strategies include compounds that inhibit bacterial pathogenicity and stubborn growth (21–23).

Sortase, a bacterial membrane enzyme, anchors a suite of surface proteins to the cell wall (24). In *S. aureus* and other gram-positive bacteria with low G + C contents, effective inhibitors of SrtA are likely to be effective against other gram-positive bacteria (25). The location of SrtA on the cell membrane enhances its targetability, making it an attractive focus for developing anti-infective drugs to prevent the emergence of resistance (26–28). Recently, antimicrobial agents from diverse natural sources have garnered increasing attention, with medicinal plants being particularly significant because their array of natural compounds can distinctly target various sites or pathways in bacterial pathogens, thus impeding or disrupting their pathogenic capabilities. Hence, our focus has been on targeting SrtA with compounds from an existing library of natural small molecules. Through FRET, we identified a SrtA inhibitor from the natural product PMS that demonstrated a lower IC_50_. Encouragingly, PMS did not affect *S. aureus* growth, suggesting a relatively lower chance of developing resistance. Additionally, it exhibited no significant cytotoxicity, prevented cell lysis, and did not cause melanization or death in *G. mellonella* larvae at concentrations of 25 and 50 mg/kg.

Adhesion is the first step in the infection process of gram-positive bacteria. In *S. aureus*, various surface proteins, including common proteins such as fibronectin-binding proteins (FnbA and FnbB) and fibrinogen-binding proteins (ClfA, ClfB, and EfB), mediate adhesion (29). These proteins are crucial for evading the host’s innate immune system and adhering bacteria to host cells, particularly because they contain the LPXTG motif. SrtA anchors these LPXTG-containing surface proteins to the bacterial cell wall (30). Our study revealed that different concentrations of PMS in semen inhibited the adhesion of *S. aureus* to fibrinogen, thereby reducing host cell invasion, as confirmed by cell invasion assays. SpA, a conserved, multifunctional surface protein of *S. aureus*, has an N-terminal region comprising five tandemly arranged three-helix bundle domains that play a significant role in binding IgG and other receptors. Owing to its numerous immunosuppressive properties, SpA is a crucial determinant of the immune evasion mechanism of *S. aureus*. It binds to the Fcγ portion of IgG, covering the bacterial surface with IgG (31), thereby reducing recognition by neutrophils and evading phagocytosis (32). In our experiments, a reduction in SpA was observed in *S. aureus* treated with PMS.

Bacteria can proliferate and colonize biotic or abiotic surfaces to form biofilms, protecting them from the host immune system and antibiotic treatment and complicating existing issues of antibiotic resistance (33). Studies have shown that *S. aureus* SrtA mutants exhibit reduced biofilm formation. As expected, coincubation of *S. aureus* with PMS significantly reduced biofilm formation (34). Our initial investigations confirmed that PMS impedes SrtA activity through a noncovalent inhibition mechanism. This distinction is crucial because it suggests that the inhibitory effect of PMS can be reversible and specific. To address the concern of nonspecific binding often associated with panassay interfering compounds (PAINS), we meticulously analyzed PMS for such properties. PAINS, known for causing many false positives in screenings due to its interference with various assay components, includes compounds prone to colloidal aggregation, autofluorescence, luciferase inhibition, instability, and broad target affinity. The low efficacy of these drugs poses significant challenges in drug development (35). Our search through the SwissADME database revealed that PMS does not possess any PAINS-related structures, substantially reducing the likelihood of false positives and affirming its potential as a drug candidate. In addition, fluorescence quenching and molecular docking further confirmed the strong direct interaction between PMS and SrtA. Notably, molecular docking revealed hydrogen bonding between PMS and SrtA-ARG197. The ARG-197 site, a key enzymatic active site, is directly involved in substrate binding and catalytic cleavage, indicating that PMS likely intervenes directly at the SrtA active site.

As one of the most common pathogens in clinical settings, *S. aureus* is a frequent cause of pulmonary infections, often leading to severe complications and high morbidity and mortality rates (36). During peak influenza seasons, coinfection with prevalent influenza viruses and *S. aureus* exacerbates patient symptoms (37). Research has indicated that, compared with their wild-type counterparts, SrtA mutants exhibit reduced pathogenicity in animal infection models, highlighting the pivotal role of this enzyme in MRSA-induced pneumonia in mouse models (38). In our study, we established a mouse pneumonia model to further assess the therapeutic efficacy of PMS semen against MRSA-induced pneumonia. Owing to its high virulence and multidrug resistance, the USA300 strain significantly challenges clinical treatment regimens. Our results demonstrated that PMS effectively combated lethal pneumonia caused by *S. aureus*, significantly increasing survival rates in mice, decreasing the bacterial load in lung tissues, and mitigating pulmonary damage. This notable decrease in efficacy is likely due to the inhibition of SrtA activity in *S. aureus*, as the formation of aggregates and biofilms is essential for bacterial adaptation within pulmonary environments.

Our study, using the checkerboard method, revealed a synergistic effect between PMS and vancomycin against MRSA, significantly enhancing antibacterial efficacy. This synergy suggests that PMS may reduce the required vancomycin dosage, potentially minimizing side effects and decreasing the likelihood of resistance development. Interestingly, although this natural small molecule lacks inherent antibacterial activity, it demonstrated synergy exclusively with vancomycin and not with a range of β-lactam antibiotics, including cefoxitin, ceftriaxone sodium, latamoxef, potassium clavulanate, cefoperazone sodium, cefotaxime, penicillin V potassium, penicillin G sodium, and cefotetan. This observation suggests that PMS enhances vancomycin efficacy through mechanisms distinct from direct bacterial inhibition. A plausible explanation is that PMS modifies specific physicochemical properties of the bacterial cell wall, potentially increasing vancomycin permeability or disrupting MRSA’s resistance pathways. Conversely, PMS’s lack of synergy with β-lactams likely results from its inability to impact PBP2a, a penicillin-binding protein encoded by the mecA gene that confers MRSA’s resistance to β-lactams by enabling cell wall synthesis despite β-lactam interference. This differential activity highlights the potential of PMS as a therapeutic adjuvant in combination therapies targeting vancomycin-specific pathways, addressing the urgent need for novel strategies to combat antibiotic-resistant infections.

Our research demonstrated that the inhibition of SrtA, a key player in bacterial virulence, represents a novel approach to reduce the dependence on conventional antibiotics. The synergy between PMS and vancomycin further highlights the importance of combination therapies in improving treatment outcomes and controlling resistance. This field of research holds great promise, offering a powerful strategy to combat bacterial infections while addressing the growing challenge of antibiotic

## MATERIALS AND METHODS

### Reagents, bacterial strains, and growth conditions

The strains involved in this experiment are detailed in Table S1 and were stored at −80°C in tryptic soy broth (TSB) supplemented with 20% glycerol. Typically, bacterial cultures were incubated in brain heart infusion (BHI) broth at 37°C with shaking at 220 rpm overnight for subsequent assays. *Escherichia coli* DH5α and BL21 (DE_3_) were cultured in Luria–Bertani broth or agar at 37°C. A549 cells were maintained in 1,640 medium supplemented with 10% fetal bovine serum and 1% penicillin‒streptomycin and grown at 37°C with 5% CO_2_ for 24 h. PMS, a flavonoid compound with a purity exceeding 98%, was obtained from Letianmei, Chengdu, China. The pertinent quality inspection report is depicted in Fig. 1 of the supplementary material. The peptide substrate Abz-LPATG-Dap (Dnp)-NH_2_ was obtained from Life Tein. Polyclonal antibodies against SrtA were produced in-house. HRP-conjugated goat anti-rabbit IgG (H + L) was purchased from Proteintech (Cat No. SA00001-2; Wuhan, China). Cefoxitin, ceftriaxone sodium, latamoxef, potassium clavulanate, vancomycin, cefoperazone sodium, cefotaxime, penicillin V potassium, penicillin G sodium, and cefotetan, all with purities greater than 99%, were purchased from Aladdin Biochemical.

### Expression and purification of the SrtA protein

For gene cloning, the prokaryotic expression vector pET-28a was utilized. The pET-28a::*srtA* plasmid was constructed using genomic DNA from *S. aureus* USA300 as a template, and the primers used are listed in Table S2. The SrtA gene fragment, which included specific restriction sites, was amplified via polymerase chain reaction (PCR). The PCR products were subsequently digested with the *NdeI* and *BamHI* enzymes and ligated into the pET-28a vector to generate the pET28a-srtA plasmid. This recombinant plasmid was subsequently transformed into *E. coli* DH5α via heat shock. Following sequencing confirmation, the plasmid was transferred similarly to *E. coli* Bl21 host cells. Once the *E. coli* culture harboring the recombinant plasmid reached an optical density (OD_600_) of approximately 0.8, isopropyl β-d-1-thiogalactopyranoside (IPTG, 0.5 mM, ST098, Beyotime, Beijing, China) was added to induce the expression of the SrtA protein. The protein was then purified via a nickel column (BeyoGold His-tag Purification Resin, Beyotime, Beijing, China), with impurities eluted via a low-concentration imidazole solution, followed by elution of the SrtA protein via a high-concentration imidazole solution.

### Expression and purification of the SrtA mutant protein

The construction of the *E. coli* pET28a::*srtA* mutant strains (A92G, A104G, V168A, K175A, and R197A) utilized the pET-28a-SrtAΔ_N59_ plasmid as a template. The primer pairs used for generating these mutations are listed in Table S2. This mutagenesis process was conducted in accordance with the specifications of the Mut Express II Fast Mutagenesis Kit V2 (Vazyme Biotech, Nanjing, China). PCR amplification was performed under the following conditions: initial denaturation at 98°C for 10 min, followed by 35 cycles of denaturation at 98°C for 30 s, annealing at 60°C for 30 s, and extension at 72°C for 20 s. A final extension step was conducted at 72°C for 10 min. The successfully constructed plasmids were then transformed into *E. coli* BL21 cells. The purification methods used for the SrtA mutant strains were identical to those used for the wild-type SrtA strains.

### Screening for SrtA inhibitors

The screening for SrtA inhibitors commenced with the preparation of a binding buffer, ensuring a final volume of 100 µL per well. Binding buffer containing 1% recombinant SrtA Δ_N59_ protein (4 µM) was added to each well, providing the necessary enzyme for the experiment. The traditional Chinese medicine monomeric compounds were subsequently introduced into the wells at gradient concentrations (64 µg/mL) and incubated at 37°C for 1 h to evaluate their inhibitory effects on SrtA. Next, a synthetic substrate peptide (10 µM) was added to each well, and the plates were incubated for 20 min at 37°C in the dark to facilitate interactions with SrtA. The fluorescence intensity of the reaction mixture was measured via a fluorometric plate reader, adjusting the gain to ensure that the enzyme activity was between 900 and 1,100. The SrtA activity inhibition rates for each sample were calculated via a specific formula. Inhibition rate = 100% × (*C* − *T*)/*C*, where *C* represents the fluorescence value of the untreated group and *T* represents the fluorescence value of the experimental group. An inhibition rate greater than 60% was considered indicative of a potential SrtA inhibitor.

### Screening for PAINS using SwissADME

The screening process for panassay interference compounds (PAINS) was performed via the SwissADME database (http://www.swissadme.ch/index.php). First, the structure of the PMS was uploaded to the SwissADME platform. The PAINS filter was applied to identify any potential interference patterns.

#### SrtA activity measurement

To assess SrtA enzyme activity, a fluorescence-based assay was conducted. The assay mixture, consisting of various concentrations of the test compound ranging from 0.5 to 256 µg/mL, was prepared in a total volume of 100 µL. This mixture included SrtA at a final concentration of 4 µM, and the reaction was allowed to proceed for 1 h at 37°C. Following this incubation, a fluorescent substrate [Abz-LPATG-Dap (Dnp)-NH_2_] was added to the mixture to achieve a final concentration of 10 µM. The reaction mixture was further incubated for 20 min to enable substrate processing by SrtA. The fluorescence intensity, which is indicative of SrtA activity, was then measured using specific excitation and emission wavelengths of 309 nm and 420 nm, respectively. The inhibition efficacy of the test compounds was quantified via the use of GraphPad Prism 8.0 software by calculating the percentage inhibition at each concentration and constructing a dose‒response curve to determine the IC_50_ value.

### Minimum inhibitory concentration

To evaluate the MIC, PMS was incrementally diluted in a twofold series, ranging from 1 to 512 µg/mL, in cation-adjusted Mueller–Hinton broth (CAMHB) broth. These dilutions were then added to a 96-well culture plate, with each well receiving 100 µL. To this mixture, a standardized inoculum of *S. aureus* USA300 (1 × 10^6^ CFU) was added, and the culture was incubated at 37°C for 16 h. A control setup comprising *S. aureus* without PMS intervention was included as a negative control, while wells containing only the medium served as the blank control. The determination of MICs was based on two criteria: absorbance at 600 nm measured via a microplate reader and visual observation of the lowest concentration at which bacterial growth was not macroscopically evident.

### Growth curves

To monitor growth kinetics, overnight *S. aureus* cultures were diluted at a ratio of 1:100 in TSB medium, with some wells receiving PMS. These cultures were then agitated at 220 rpm at 37°C. The absorbance at 600 nm was systematically recorded at predetermined intervals over a 24 h period, and a microplate reader was used to track the growth trajectory of the bacteria.

### Cytotoxicity assay

Cell toxicity was evaluated via the Cell Counting Kit-8 (CCK-8) from TransGen Biotech. In brief, A549, HepG2, or 293T cells, at a density of 5 × 10^4^ cells/100 µL, were cultured in 96-well plates and incubated at 37°C in a 5% CO_2_ atmosphere for 24 h. The old medium was subsequently replaced with fresh medium infused with various concentrations of PMS (ranging from 0 to 64 µg/mL) or dimethyl sulfoxide (DMSO, D12345, Invitrogen, USA). Following a further 24 h incubation, each well received 10 µL of CCK-8 solution and was incubated for 1–4 h. The absorbance at 450 nm, corresponding to the dye generated by the substrate conversion of the CCK-8 kit, was measured via a microplate reader to assess cell viability. This procedure was replicated a minimum of three times. The relationship between the PMS concentration and cell viability was graphically represented via GraphPad Prism 8.0.

### Fibrinogen-binding assay

An adhesion assay was conducted in a 96-well plate to measure the binding of *S. aureus* to fibrinogen. Each well was coated with 100 µL of bovine fibrinogen (Fg, 20 µg/mL) at 4°C overnight. Subsequently, overnight cultures of *S. aureus* (cultured in BHI medium at 37°C with shaking at 200 rpm) were diluted at a 1:100 ratio and incubated with varying concentrations of PMS (0–64 µg/mL). After overnight incubation, the supernatant was discarded, and each well was stained with 40 µL of crystal violet (12.5 g/L, C110703-25 g, Aladdin, China) for 20 min. Excess dye was removed by washing with PBS, the mixture was eluted with 200 µL of absolute ethanol, and the absorbance was measured at a wavelength of 595 nm. The controls included Δ*srtA* and BHI media as positive and blank controls, respectively.

### Crystal violet biofilm assay

Crystal violet staining was performed using a 96-well polystyrene plate. The overnight bacterial cultures were diluted to a ratio of 1:100 in BHI medium enriched with 0.5% glucose. To each well, 200 µL of this bacterial mixture was added, along with varying concentrations of PMS (0–64 µg/mL), and the mixture was subsequently incubated at 37°C for 18 h. After incubation, the developed biofilms were stained for 15 min with a 0.1% (wt/vol) crystal violet solution. The wells were then emptied of excess stain and washed three times with sterile distilled water to remove any unbound dye. The quantitative analysis of the biofilm was carried out by dissolving the crystal violet in isopropyl alcohol and measuring the absorbance at 595 nm.

### FITC-IgG binding to staphylococci

*S. aureus* USA300 was cultured overnight and then diluted 1:100 in TSB media supplemented with either PMS or DMSO, after which the cultures were grown to an OD_600_ of 1.0, with Δ*srtA* serving as a positive control. The bacteria were then centrifuged and resuspended in 0.5% bovine serum albumin (BSA), followed by three PBS washes. The samples were subsequently incubated with FITC-labeled rabbit anti-goat IgG (#SA00003-4, Proteintech, China) for 1 h at room temperature. After additional washes, flow cytometry was utilized to assess the amount of bound IgG on the bacteria by analyzing the fluorescence intensity of 10,000 cells.

### Cellular invasion assay

A549 cells were seeded at a density of 2 × 10⁴ cells per well in 24-well plates and incubated overnight at 37°C with 5% CO₂. Overnight cultures of *S. aureus* USA300 were diluted 100-fold and cocultured with varying concentrations of PMS (0–64 µg/mL) at 37°C with shaking at 200 rpm until the culture reached an OD₆₀₀ of 1.0. The bacterial cultures were centrifuged, and the bacterial pellets were resuspended in PBS. A bacterial suspension was added directly to the A549 cell monolayers at a multiplicity of infection (MOI) of 100 and incubated for 2 h at 37°C. Following three washes with PBS, the cells were incubated with 1,604 medium containing 300 µg/mL gentamicin for 1 h to kill the extracellular bacteria. Afterward, the A549 cells were lysed with 1% Triton X-100 for 30 min, and the lysates were serially diluted in PBS. The diluted lysates were plated on tryptic soy agar (TSA) for colony-forming unit (CFU) enumeration via the direct colony counting method.

### Live/dead cell assay

*S. aureus* USA300 was cultured until the OD_600_ reached 0.5, followed by washing with PBS and suspension in fresh Dulbecco’s modified Eagle medium (DMEM). Various concentrations of PMS (0–64 µg/mL) were then added to the bacterial suspension. A549 cells were cultured in DMEM supplemented with 10% fetal bovine serum and seeded into 24-well plates at a density of 1 × 10⁵ cells per well. After the cells were washed twice with PBS, 500 µL of the bacterial suspension was added to each well. The cells were incubated at 37°C for 4 h. Following incubation, a calcein-AM/propidium iodide (PI) cytotoxicity detection kit (L6037M; US Everbright, Suzhou, China) was used to assess the protective effects of PMS on cellular damage, and observations were made under a fluorescence microscope.

### LDH cytotoxicity assay

The supernatants of the A549 cell cultures were collected after the live/dead assay, and the levels of LDH were quantified via an LDH cytotoxicity assay kit (C0016; Beyotime, Shanghai, China) to further evaluate cell damage.

### Western blot

For the Western blot analysis of SrtA in *S. aureus* USA300, cultures diluted at a 1:100 ratio were treated with varying concentrations of PMS (0–64 µg/mL) and incubated for 12 h. After incubation, the bacterial cells were harvested, resuspended in PBS supplemented with lysostaphin, and sonicated. The supernatant containing SrtA was collected for further analysis. Proteins were separated via 10% SDS‒PAGE and transferred to polyvinylidene fluoride (PVDF) membranes. The membranes were blocked with 3% BSA to prevent nonspecific binding, followed by incubation with a rabbit anti-SrtA antibody and a horseradish peroxidase (HRP)-labeled secondary antibody. Protein bands were detected through chemiluminescence, and band intensities were quantified via ImageJ 1.52a software.

### Reversible inhibition of SrtA

In line with previous reports (39), the reversible inhibition of SrtA was assessed. At room temperature, 100 µL of purified SrtA was incubated with PMS at a final concentration 10 times greater than the IC_50_ for 1 h, followed by dilution with 9.9 mL of reaction buffer (6.057 g Tris-HCl, 0.555 g CaCl2, and 8.775 g NaCl dissolved in 1,000 mL of deionized water). Subsequently, 10 µL of the fluorescent substrate peptide (Abz-LPATG-Dap (Dnp)-NH_2_, 1 mg/mL) was mixed with 190 µL of the dilution. Fluorescence intensity measurements were conducted at excitation and emission wavelengths of 309 nm and 420 nm, respectively.

### Fluorescence quenching assay

The binding affinity, expressed as the binding constant (*K*_A_), between PMS and the SrtA enzyme was quantitatively assessed via an advanced fluorescence quenching assay (40). The assay commenced with the precise preparation of a reaction mixture containing 20 µL of PMS and 1,980 μL of highly purified SrtA, yielding final concentrations ranging from 0 to 45 µg/mL. A specific concentration of SrtA (5 µM) was used to ensure optimal interaction conditions. Fluorescence measurements were meticulously carried out by setting the excitation wavelength at 280 nm, with a narrow bandpass of 5 nm, and capturing emission spectra from 260 to 400 nm. A state-of-the-art fluorescence spectrophotometer was used to record the emission spectra of the mixtures. The resulting data were carefully plotted on a Stern–Volmer plot, depicting F0/F against [Q]. Linear regression analysis was then applied to calculate the quenching constant accurately, providing insights into the molecular interactions between PMS and SrtA.

### Molecular docking studies for interaction analysis

For a deeper understanding of the molecular interactions, molecular docking studies were conducted. The three-dimensional structural data for PMS were sourced from the PubChem database, ensuring the use of the most updated and accurate molecular model. This structure was computationally docked with the crystal structure of the SrtA enzyme (PDB ID: 1T2P), allowing the exploration of potential binding sites and interaction patterns. The docking parameters were meticulously set, with an exhaustiveness level of 8 to ensure thorough sampling of possible orientations. The grid box for the docking simulations was carefully adjusted to the following specific coordinates to precisely encapsulate the active site of SrtA: center_x = −30.329, center_y = −19.713, center_z = −0.456, dimensions size_x = 41.25, size_y = 45.0, and size_z = 47.25. The docking process was performed via the advanced capabilities of AutoDock Vina1.57, and the resulting docked complexes were visualized and analyzed via PyMOL 2.3, which provided a detailed representation of the molecular interactions.

### MRSA infections in *G. mellonella* larvae

In the *G. mellonella* infection assay, the effectiveness of PMS against *S. aureus* was evaluated using *G. mellonella* larvae. These larvae were divided into five groups: a USA300 infection group, an untreated control group, a Δ*srtA* infection group, and two PMS-treated groups with varying dosages (25 mg/kg and 50 mg/kg), with each group comprising 10 larvae. For infection, larvae received a 10 µL injection of an MRSA suspension (5 × 10^6^ CFU/mL) into their leftmost proleg. Treatments commenced 1 h postinoculation, with the PMS groups receiving designated doses and the control group remaining untreated. The larvae were kept in a stable 37°C environment, and survival was monitored every 12 for 120 h. In parallel, for colony count assessments, methodologies from the survival study were employed. Larvae were collected 48 h after infection, sterilized, homogenized, and cultured on TSA at 37°C for 24 h for colony enumeration. This approach established a sensitivity threshold of 100 CFU/mL for larval homogenates. All experiments were repeated a minimum of three times.

### Murine model of pneumonia

To establish a murine model of pneumonia, eight-week-old inbred C57BL/6J male mice, each weighing 20–22 g, were utilized. The methodology commenced with the preparation of *S. aureus* USA300 and Δ*srtA*. These were initially cultured overnight, subsequently diluted 1:100 in sterile TSB broth, and allowed to grow until they reached an OD_600_ value of 1.0. The bacterial cultures were then harvested via low-speed centrifugation, washed three times in PBS, and finally resuspended in saline.

For experimental infection, the mice were anesthetized with isoflurane gas and intranasally inoculated with 2 × 10^8^ CFU (30 µL) of the prepared *S. aureus* suspension. Postinfection, a single dose of 25 or 50 mg/kg PMS was administered subcutaneously to each mouse, followed by subsequent doses every 12 h. Survival rates were monitored at intervals from 12 to 96 h after infection.

To assess the therapeutic efficacy of PMS, each mouse in the infection group was administered 1 × 10^8^ CFU (30 µL) of *S. aureus*, followed by administration of the medication in the same manner. The mice were euthanized 48 h postinfection. Lung tissues were dissected for both bacterial load assessment and histopathological analysis. The latter involved staining with hematoxylin and eosin (H&E) for detailed microscopic examination of pulmonary tissue architecture and inflammatory infiltration. Furthermore, this study included an analysis of lung wet‒dry weight ratios to further understand the impact on pulmonary tissues. This method involves weighing the excised lungs immediately after extraction to obtain the wet weight. The lungs were subsequently dried in an oven at a controlled temperature until a consistent dry weight was achieved. The dry weight was then recorded, and the wet‒dry weight ratio was calculated to assess the extent of pulmonary edema, which is a critical indicator of lung injury and inflammation.

### Checkerboard assay

A two-dimensional broth microdilution checkerboard assay was employed in 96-well plates to investigate the interaction between two compounds. The MIC values of cefoxitin, ceftriaxone sodium, latamoxef, potassium clavulanate, vancomycin, cefoperazone sodium, cefotaxime, penicillin V potassium, penicillin G sodium, and cefotetan against USA300 are presented in Table S3. In this method, one antibiotic is serially diluted along the vertical axis, while the second compound is diluted along the horizontal axis. A bacterial suspension, with a final inoculum concentration of 0.5 × 10^6^ to 1 × 10^6^ CFU/mL, was added to each well. Following a 20 h incubation at 37°C, the MIC for each antibiotic was determined as the lowest concentration that visibly inhibited bacterial growth. The interaction between the antibiotics was assessed through the fractional inhibitory concentration (FIC) indices. The FIC index was calculated by summing the FIC values for each drug: FIC = FIC A + FIC B, where FIC A represents the MIC of drug A in combination relative to its MIC alone, and FIC B represents the MIC of drug B in combination relative to its MIC alone (41).

### Statistical analysis

Each study was independently repeated three times. Unless otherwise specified, the results are expressed as the means ± SD. A *P* value less than 0.05 was considered to indicate statistical significance. All the statistical analyses were conducted via GraphPad Prism 8.0. *T* tests were used to compare two groups, while survival rates were analyzed via the log-rank test. Additionally, one-way ANOVA was employed for comparisons involving more than two groups.

## Data Availability

The data sets generated during and/or analyzed during the current study are available from the corresponding author upon reasonable request.

## SUPPLEMENTAL MATERIAL

Supplemental material aem.01804-24-s0001.docx Figures S1 and S2; Tables S1 to S3.

ASM does not own the copyrights to Supplemental Material that may be linked to, or accessed through, an article. The authors have granted ASM a non-exclusive, world-wide license to publish the Supplemental Material files. Please contact the corresponding author directly for reuse.
