# Supplementary material for: Novel inhibition of Staphylococcus aureus sortase A by plantamajoside: implications for controlling multidrug-resistant infections
Source: Appl Environ Microbiol. 2024 Dec 31;91(1):e01804-24. doi: 10.1128/aem.01804-24 (PMC11784452; doi:10.1128/aem.01804-24)
Supplement: Supplemental material — Figures S1 and S2; Tables S1 to S3. [file aem.01804-24-s0001.docx]

**Supplemental Information**

**Novel Inhibition of *Staphylococcus aureus* Sortase A by PMS: Implications for Controlling Multidrug-Resistant Infections**

**Yujia Chen^1^, Wei Li^1^, Li Wang^2^, Bingmei Wang^2^, Jian Suo^1*^**

^1^ Department of Gastrocolorectal Surgery, General Surgery Center, The First Hospital of Jilin University, Changchun, China.

^2^ Clinical Medical College, Changchun University of Chinese Medicine, Changchun, China.

*Correspondence: Jian Suo, suojian0066@126.com

**Table of contents**

Figure S1. Quality assessment report for PMS.

Figure S2. Electrophoretic profiling of the purification progression for the SrtA protein via SDS‒PAGE.

Figure S3. Comprehensive toxicity analysis of PMS in *Galleria mellonella*.

Table S1. Strains used in this study.

Table S2. Oligonucleotide primers used in this study.

Table S3. The antibacterial effect of antibiotics combined with PMS against *S. aureus.*

**Figure S1.** **Quality assessment report for PMS.**

**A**


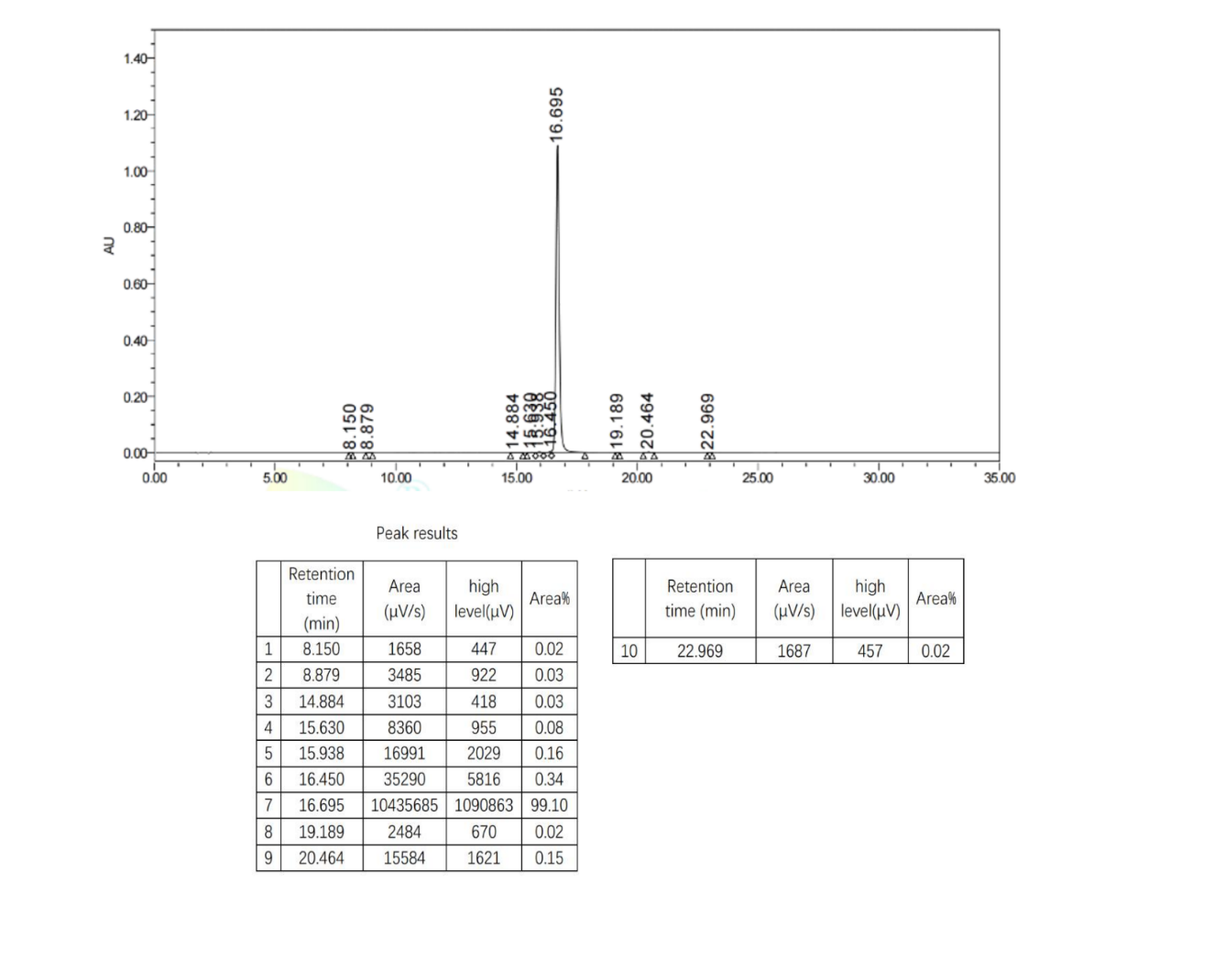


**B**


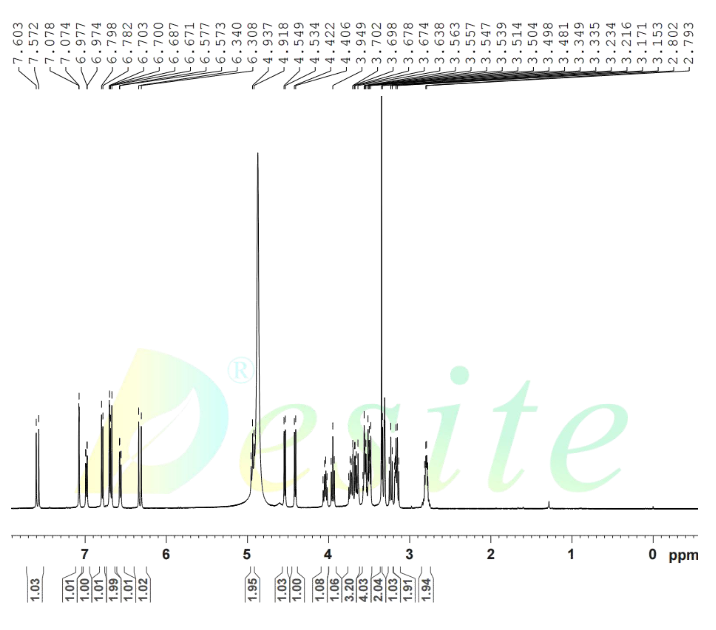


Figure S1. PMS Quality Analysis Report. (A) High-Performance Liquid Chromatography (HPLC) Profile - Chromatographic evaluation confirming the purity of PMS, quantified at 99.16%. (B) Proton nuclear magnetic resonance (^1^H-NMR) spectral analysis - Detailed analysis of PMS using 1H-NMR spectroscopy.

**Figure S2.** Electrophoretic profiling of the purification progression for the SrtA protein via SDS‒PAGE.

**
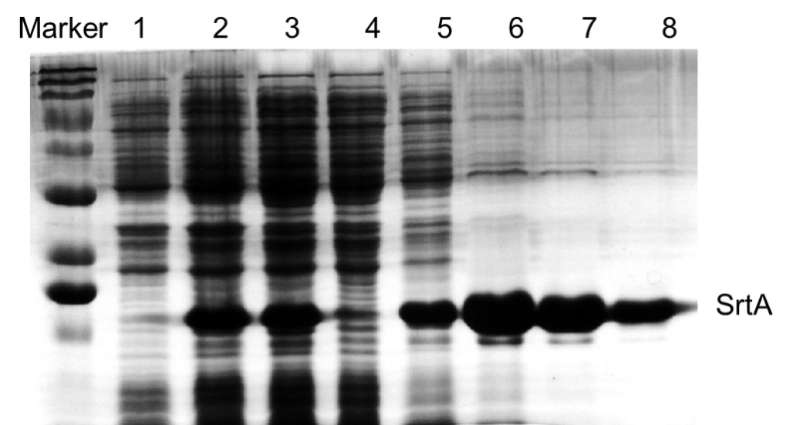
**

Figure S2. Analysis of SrtA Protein Purification from BL21 (DE3) pET28a::SrtA by SDS‒PAGE. Lanes 1 and 2 demonstrate the expression of BL21 (DE3) pET28a::SrtA before and after induction with IPTG, respectively. Lane 3 contains the supernatant of the lysate from sonicated transformed BL21(DE3) cells. Lane 4 highlights nonspecific proteins eluted at a low imidazole concentration (20 mM). Proteins collected at a moderate imidazole concentration (50 mM) are shown in lane 5, while lanes 6-8 display the target SrtA protein purified under stringent conditions using 100, 200, and 400 mM imidazole, respectively.

**Figure S3.** Comprehensive toxicity analysis of PMS in *Galleria mellonella*.


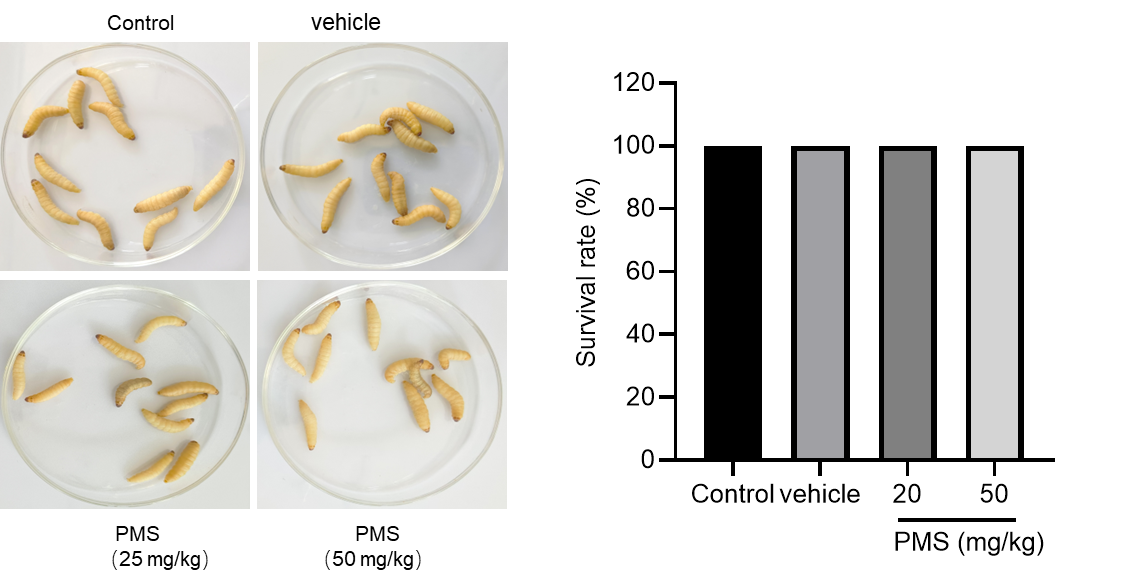


Figure S3. Comprehensive toxicity analysis of PMS in *Galleria mellonella*. This figure provides a detailed evaluation of the biological impact of administering PMS at dosages of 25 mg/kg and 50 mg/kg on *Galleria mellonella* larvae. It specifically examines two critical parameters: the degree of melanization and the overall survival rate of the larvae. The data illustrate that the administered dosages do not induce significant alterations in melanization or compromise larval survival compared to the control group. This lack of adverse effects, particularly in terms of physiological responses and mortality rates, implies the relative safety of PMS at these concentrations.

**Table S1. Strains used in this study**

| **Strains** | **Source** |
| --- | --- |
| MRSA  pET28a::*SrtA*- Bl21(DE_3_)  USA300-Δ*SrtA*  pET28a::s*rtA* (A92G) -Bl21(DE_3_)  pET28a::s*rtA* (A104G)-Bl21(DE_3_)  pET28a::s*rtA* (K175A)-Bl21(DE_3_)  pET28a::s*rtA* (R197A)-Bl21(DE_3_) | ATCC^®^ BAA-1717™ (USA300-HOU-MR)  This study  This study  This study  This study  This study  This study |

ATCC, American Type Culture Collection; MRSA, methicillin-resistant *Staphylococcus aureus*

**Table S2 Oligonucleotide primers used in this study.**

| **Primers** | **Nucleotide sequence (5’→ 3’)** | **Purpose** |
| --- | --- | --- |
| *srtA* | GGGAATTCCATATGCAAGCTAAACCTCAAATTCCG | PCR |
|  | CGCGGATCCTTATTTGACTTCTGTAGCTACAAAGA |  |
| A92G | CCAGTATATCCAGGACCAGGCACACCTGAACAATTAAATAG | qPCR |
|  | CTATTTAATTGTTCAGGTGTGCCTGGTCCTGGATATACTGG |  |
| A104G | GGTGTAAGCTTTGGCGAAGAAAATGAATC | qPCR |
|  | GATTCATTTTCTTCGCCAAAGCTTACACC |  |
| V168A | CTACAGATGTAGGAGCCCTAGATGAACAAAAAG | qPCR |
|  | CTTTTTGTTCATCTAGGGCTCCTACATCTGTAG |  |
| K175A | GATGAACAAAAAGGTGCCGATAAACAATTAAC | qPCR |
|  | GTTAATTGTTTATCGGCACCTTTTTGTTCATC |  |
| R197A | GTTTGGGAAAAAGCTAAAATCTTTG | qPCR |
|  | CAAAGATTTTAGCTTTTTCCCAAAC |  |

**Table S3. The antibacterial effect of antibiotics combined with PMS against *S. aureus***

| **Antibiotics** | **MIC_（μg/mL）_** | **FIC**  **_antibiotics_** | **MIC _PMS_**  **_（μg/mL）_** | **FIC**  **_PMS_** | **FICI** |
| --- | --- | --- | --- | --- | --- |
| Cefoxitin | 16 | 0.5 | 512 | 0.125 | 0.625 |
| Ceftriaxone Sodium | 32 | 0.5 | 512 | 0.125 | 0.625 |
| Latamoxef | 8 | 0.5 | 512 | 0.125 | 0.625 |
| Potassium clavulanate | 128 | 1 | 512 | 0.125 | 1.125 |
| Vancomycin | 4 | 0.25 | 512 | 0.125 | 0.375 |
| Cefoperazone Sodium | 16 | 1 | 512 | 0.125 | 1.125 |
| Cefotaxime | 16 | 1 | 512 | 0.125 | 1.125 |
| Penicillin V Potassium | 8 | 0.5 | 512 | 0.125 | 0.625 |
| Penicillin G Sodium | 512 | 0.5 | 512 | 0.125 | 0.625 |
| Cefotetan | 32 | 1 | 512 | 0.125 | 1.125 |
